# Probabilistic modeling of the evolution of gene synteny within reconciled phylogenies

**DOI:** 10.1186/1471-2105-16-S14-S5

**Published:** 2015-10-02

**Authors:** Magali Semeria, Eric Tannier, Laurent Guéguen

**Affiliations:** 1 Laboratoire de Biométrie et Biologie Évolutive UMR CNRS 5558, Université Claude Bernard Lyon 1, 43 boulevard du 11 novembre 1918, 69622 Villeurbanne, France; 2 INRIA Grenoble Rhône-Alpes, 655 avenue de l'Europe, 38330 Montbonnot, France

**Keywords:** genome rearrangements, gene order, gene tree reconciliation, Bio++

## Abstract

**Background:**

Most models of genome evolution concern either genetic sequences, gene content or gene order. They sometimes integrate two of the three levels, but rarely the three of them. Probabilistic models of gene order evolution usually have to assume constant gene content or adopt a presence/absence coding of gene neighborhoods which is blind to complex events modifying gene content.

**Results:**

We propose a probabilistic evolutionary model for gene neighborhoods, allowing genes to be inserted, duplicated or lost. It uses reconciled phylogenies, which integrate sequence and gene content evolution. We are then able to optimize parameters such as phylogeny branch lengths, or probabilistic laws depicting the diversity of susceptibility of syntenic regions to rearrangements. We reconstruct a structure for ancestral genomes by optimizing a likelihood, keeping track of all evolutionary events at the level of gene content and gene synteny. Ancestral syntenies are associated with a probability of presence.

We implemented the model with the restriction that at most one gene duplication separates two gene speciations in reconciled gene trees. We reconstruct ancestral syntenies on a set of 12 *drosophila *genomes, and compare the evolutionary rates along the branches and along the sites. We compare with a parsimony method and find a significant number of results not supported by the posterior probability. The model is implemented in the Bio++ library. It thus benefits from and enriches the classical models and methods for molecular evolution.

## Background

Genomes evolve through processes that modify their content and organization at different scales, ranging from substitutions, insertions or deletions of single nucleotides to large scale chromosomal rearrangements. Extant genomes are the result of a combination of many such processes, which makes it difficult to reconstruct the big picture of genome evolution. Instead, most models and methods focus on one scale and use only one kind of data, such as gene orders or sequence alignments.

Models based on sequence alignments were first developed in the 1960's and underwent steady development until reaching a high level of complexity [1]. In a recent development, they have been extended to include gene content, modeling duplications, losses and transfers of genes with reconciliation methods [2,3]. Reconciled gene trees account for evolutionary events at both the sequence level and the gene family level. They thus yield a better representation of genome evolution and pave the way for approaches integrating other levels of information [4,5].

In parallel extant gene orders have long been used to infer evolutionary relationships between organisms and to reconstruct ancestral genomes [6-8]. Although the early stages of their development were computational challenges, methods based on gene orders gradually overcame theoretical and computational constraints so that they can now handle unequal gene content, multi-chromosomal genomes, whole genome duplications and dozens of genomes with large amounts of genes [9-11], and can be inserted into probabilistic frameworks [12-17].

All ingredients are present to integrate gene order and sequence evolution models, yet this leap has not been taken, mostly because of computational issues. Reconstructing gene order histories is often hard [18]. A computational solution to reconstruct gene orders and scale up with the size of datasets is to see a genome as a set of independently evolving adjacencies, *i.e*. the links between consecutive genes [19]. One can reconstruct ancestral gene orders following three main steps:

• Group potentially homologous adjacencies (they connect homologous pairs of genes)

• For each group, reconstruct the common history of adjacencies, by recovering ancestral ones

• Assemble the ancestral adjacencies in each ancestral species to obtain ancestral chromosomes

The assumption that adjacencies evolve independently allows quick computations at the second step: the size of the data can be an order of magnitude larger than without the assumption. But an optimization assembly step is required because of possible conflicts between adjacencies wrongly assumed independent [20].

Another difficulty is the integration of gene content dynamics. Often probabilistic solutions are limited to invariable gene content [12-14]. A solution is to encode altogether the presence and absence of genes and adjacencies as binary characters and use a binary sequence evolution model [15,16], but it lacks an evolutionary model of gene content and order dynamics. Gene profiles [21] or reconciled gene trees [22,10] are more promising for integration with sequence evolution models. They were mainly used with parsimony methods to reconstruct ancestral adjacencies, which makes it difficult to combine with a model at a different scale.

We propose a probabilistic model of adjacency evolution accounting for gene duplications and losses, using extant gene orders and reconciled gene trees. We base on the parsimony algorithm of DeCo [10] that we adapt to Felsenstein's maximum likelihood algorithm [1] with a birth/death process that models the evolution of adjacencies. We compute the most likely adjacencies in ancestral genomes and the quantity of gains and losses of adjacencies in all the branches of a species trees, thus providing an insight into the dynamics of rearrangements in these lineages. The model is implemented in Bio++ [23], the present form allowing at most one duplication node between two speciation nodes in gene trees. We compute the likelihood of gene orders in a set of 12 drosophila whose genomes are annotated in the Ensembl Metazoa [24] database. We optimize branch lengths in a species phylogeny and construct ancestral genomes. We compare the results with a parsimony approach, showing that while most adjacencies inferred by parsimony have a good probability, a non negligible proportion (> 11%) are not supported (posterior probability < 0.5).

## Methods

### Input

#### Species tree

A rooted species tree is a binary tree that describes the evolutionary relationships between organisms. The leaves of the tree are available species, internal nodes are ancestral species. The species tree has branch lengths indicating the quantity of expected evolution. Branch lengths can be also estimated as an output.

#### Adjacencies

An ordered set of genes is represented by a set of *adjacencies*, which are pairs of consecutive genes. For example, a genome *A *containing the sequence of genes *a*_1 _− *a*_2 _− *a*_3 _− *a*_4 _contains adjacencies *a*_1_*a*_2_, *a*_2_*a*_3_, and *a*_3_*a*_4_. Adjacencies are not oriented, meaning that *a*_1_*a*_2 _is equivalent to *a*_2_*a*_1_.

#### Gene trees

Genes are grouped into homologous families across genomes. The evolutionary history of each family is represented by a rooted gene tree. Gene trees are reconciled with the species tree (see precomputation below).

### Principle

**Figure 1:**
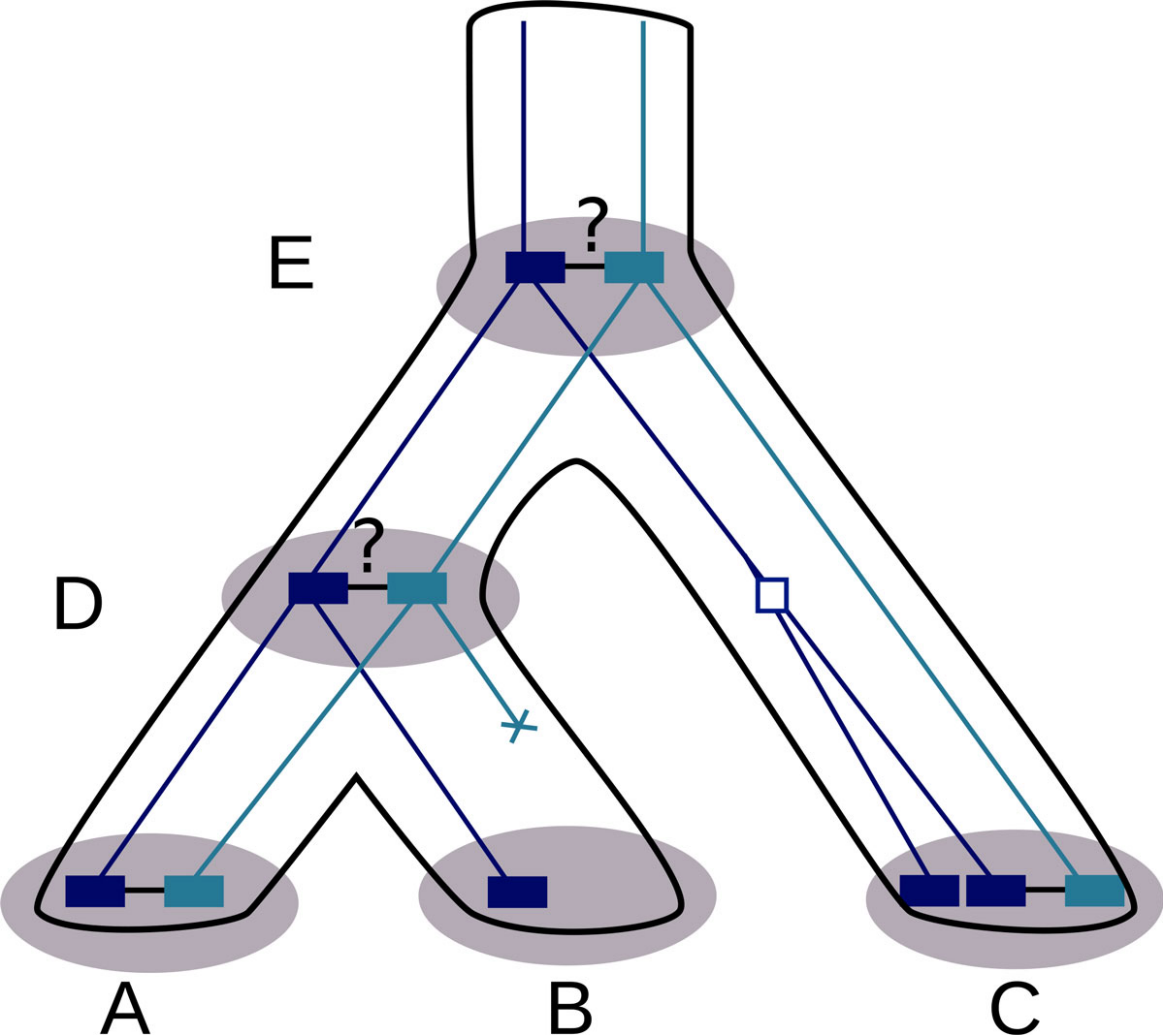
**Principle of the method**. Given two gene trees (dark blue tree and light blue tree) reconciled within a species tree (black tree), and sharing adjacencies in some extant species (species A and C), we reconstruct hypothetical ancestral adjacencies (in species D and E) using a model of evolution and maximum likelihood algorithm. Our method allows for losses (cross in light blue tree between species B and species D), and duplications (empty square in dark blue tree) of genes.

The principle is illustrated on Figure 1. It consists in reconstructing hypothetical ancestral adjacencies, modeling the evolution of adjacencies, computing a maximum likelihood of the model given the data, and computing the *a posteriori *probability of presence for each ancestral adjacency.

In this section, we give an overview of the main steps in our method. All these steps are detailed in the following sections, except the precomputation, for which we refer to [10].

• Precomputation: gene trees are reconciled with the species tree in order to minimize the number of duplications and losses (using DeCo [10]). It consists in annotating each internal node by an ancestral gene, together with the species it belongs to, and the evolutionary event (speciation, duplication, loss) taking place at the bifurcation. This determines a set of ancestral genes for all ancestral species. Gene losses are also annotated in the trees.

• Classify extant adjacencies so that every class can be handled independently. Inside each class, two gene families with two trees are involved and all adjacencies have an extremity in each family.

• For each selected pair of gene families, construct a tree, called the *tree of possible adjacencies*. Its nodes are all the couples of nodes from each gene tree, which are in the same extant or ancestral species (the speciation nodes), plus some duplication nodes; the leaves are labeled with the pattern of presence/absence of the possible adjacencies in the data.

• Compute, between successive nodes of this tree, the probability of presence or absence of the adjacency using the model of evolution described below.

• Compute the likelihood of the adjacency given the observed adjacencies.

• Compute *a posteriori *probabilities of presence of ancestral adjacencies.

The likelihood computation for one adjacency tree allows to obtain a likelihood for the whole dataset by multiplying all likelihoods, considered as independent, and to optimize parameters. These can concern branch lengths on the species tree, or a law of differential fragility for different genome sites, modeling different susceptibility to rearrangements among chromosomal regions [25,26].

### Adjacency classes

We first reduce the problem to two gene trees, without loss of generality, by classifying adjacencies. Reconciled gene trees define ancestral genes of ancestral species. A necessary condition for an adjacency *i*_1_*i*_2 _to be an ancestor of *a*_1_*a*_2 _is that *i*_1 _is an ancestor of *a*_1 _and *i*_2 _an ancestor of *a*_2_. By the same idea a necessary condition for adjacencies *a*_1_*a*_2 _and *b*_1_*b*_2 _to be homologous is that there is a common ancestor *i*_1 _of *a*_1 _and *b*_1_, and a common ancestor *i*_2 _of *a*_2 _and *b*_2_, such that *i*_1 _and *i*_2 _are in the same species. This condition for homology is an equivalence relation on all extant adjacencies, which can be clustered and treated by equivalence classes of homology. To a class we can associate *i*_1 _and *i*_2 _the most ancient distinct common ancestors of all adjacency extremities in the class. So every adjacency in the class has an extremity which is a descendant of *i*_1 _and an extremity which is a descendant of *i*_2_. Without loss of generality we can work with the two sub-trees rooted at *i*_1 _and *i*_2_.

### Trees of possible adjacencies

We now suppose that we have *G*_1 _and *G*_2 _two reconciled gene trees with some leaves of *G*_1 _involved in adjacencies with some leaves of *G*_2_. Each node *n *in *G*_1 _and *G*_2 _is annotated with an event (speciation, duplication, loss) and a species *S*(*n*). Take each pair of nodes *i*_1_*i*_2_, where *i*_1 _and *i*_2 _are speciation nodes associated with the same ancestral species *s, i*_1 _∈ *G*_1 _and *i*_2 _∈ *G*_2_. Since *S*(*i*_1_) = *S*(*i*_2_) and adjacencies exist between leaves of *G*_1 _and leaves of *G*_2_, *i*_1_*i*_2 _is called a *possible adjacency*.

All possible adjacencies define nodes of the tree of possible adjacencies, in which duplication nodes can be added, as explained below.

If *i*_1_*i*_2 _is a possible adjacency such that *S*(*i*_1_) = *S*(*i*_2_) = *s*, let *s*_1 _and *s*_2 _be the two children of *s *in the species tree. There is a descent path in the tree of possible adjacencies from *i*_1_*i*_2 _to all possible adjacencies *j*_1_*j*_2 _in *s*_1 _such that *i*_1 _is an ancestor of *j*_1 _and *i*_2 _is an ancestor of *j*_2_, and a similar independent path from *s *to *s*_2_. If there is no duplication node between *i*_1 _and *j*_1 _and *i*_2 _and *j*_2_, then this path is a single edge. If there is at least one duplication node between *i*_1 _and *j*_1 _or *i*_2 _and *j*_2_, then the path from *i*_1_*i*_2 _to *j*_1_*j*_2 _has two edges, one between *i*_1_*i*_2 _and *d*, a new duplication node, and one from *d *to *j*_1_*j*_2_. The node *i*_1_*i*_2 _always has only two descendants, but the node *d *can have an arbitrary number, according to the number of duplications in the gene lineages.

**Figure 2:**
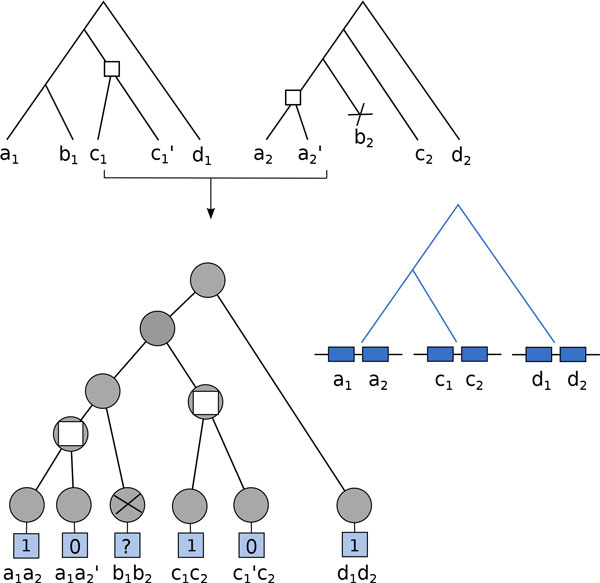
**The tree of possible adjacencies**. A tree of possible adjacencies is constructed from two reconciled gene trees (top trees). The nodes of the tree are annotated as speciation nodes (grey nodes), duplication nodes (white squares), or losses (crossed grey nodes). A binary state is attributed to each leaf according to the presence/absence pattern of the adjacency in extant species (light blue square). The true evolutionary history of the adjacency is represented on the blue tree. An adjacency exists between genes *a*_1 _and *a*_2_, *c*_1 _and *c*_2_, *d*_1 _and *d*_2_. It is absent between genes *a*_1 _and $a_{2}^{\prime }$ and genes $c_{1}^{\prime }$ and *c*_2_. If one extremity of the adjacency is lost (species B), the adjacency node is given an undefined state "?".

Loss of one or both genes involved in the adjacency in a branch leading to a species *s′ *leads to the loss of the adjacency in *s′*. In this case, a *loss leaf *of the tree of possible adjacencies is constructed. An example of construction of a tree of possible adjacencies for two reconciled gene trees is drawn in Figure 2. Once each pair of nodes *i*_1_*i*_2 _has been considered, the resulting tree is the tree of possible adjacencies for *G*_1 _and *G*_2 _on which we can apply a model of evolution.

### Model of evolution

We consider possible adjacencies as evolutionary objects in a binary alphabet. An adjacency can either be present (state 1) or absent (state 0) in a genome. The transition rate matrix for the birth/death process which describes the evolution of a binary object is:

(1) $$Q=\left(\begin{array}{cc} -\frac{\kappa +\text{1}}{\text{2}} & \frac{\kappa +\text{1}}{\text{2}}\\ \frac{\kappa +\text{1}}{\text{2}\kappa } & -\frac{\kappa +\text{1}}{\text{2}\kappa } \end{array}\right)$$

Where *κ *is the rate of 0 → 1 (gain of an adjacency) over the rate of 1 → 0 (loss of an adjacency). Probabilities of transition between two states separated by a amount *t *of time can be computed using a classical binary substitution model:

(2) $$P\left(t\right)=\left(\begin{array}{cc} \frac{\text{1}+\kappa e^{-\lambda t}}{\kappa +\text{1}} & \frac{\kappa -\kappa e^{-\lambda t}}{\kappa +\text{1}}\\ \frac{\text{1}-e^{-\lambda t}}{\kappa +\text{1}} & \frac{\kappa +e^{-\lambda rt}}{\kappa +\text{1}} \end{array}\right)$$

Where $\lambda =\frac{{\left(\kappa +\text{1}\right)}^{2}}{2\kappa }.$

In the case when there is no duplication in the two gene trees, likelihoods can be computed directly from the tree of possible adjacencies (which itself has no duplication nodes) with Felsenstein's algorithm [1].

An adjacency can be lost because of a rearrangement (1 → 0), or because at least one of the two adjacent genes is lost. In the first case, the state of the leaf in the tree of possible adjacency is simply 0. In the second case, we assign an undetermined state ? to the loss leaf in the tree of possible adjacencies to differentiate it from a loss due to a rearrangement. We do not compute probabilities of transition for branches leading to these nodes.

In the case when there are duplication nodes, we write the probabilities according to a model of evolution of adjacencies in presence of duplications: when one gene belonging to an adjacency is duplicated, the adjacency is transmitted to one of the two copies of the gene. This is always verified, whether the duplication is tandem or remote. For example, consider a gene *i*_2 _involved in an adjacency *i*_1_*i*_2 _in species *I *with a gene *i*_1_. In species *A *(descendant of species *I *), *i*_1 _has one descendant *a*_1_, whereas *i*_2 _is duplicated, giving two copies *a*_2 _and $a_{2}^{\prime }$. If the duplication is in tandem it leads to the gene order $a_{\text{1}}a_{\text{2}}a_{2}^{\prime }$, and the only adjacency conserved with *a*_1 _is *a*_1_*a*_2_. Otherwise it leads to the gene order $a_{\text{1}}a_{\text{2}}.\;.\;.\;a_{2}^{\prime }$ and again only *a*_1_*a*_2 _is conserved. Note nevertheless that the adjacency $a_{\text{1}}a_{2}^{\prime }$ can appear later in the phylogeny following a rearrangement.

Between two speciation events, we have no date for duplication events. We argue that fixing a date, for example with gene branch lengths, would be a mistake as the position of a duplication between two speciations influences the transition probabilities. Besides, the probabilistic approach means that we can account for all possible dates. Hence we compute an average transition probability for the duplicated branch over all the moments on the branch of the species where this duplication could have occurred. To do this, we integrate the transition probabilities *P*(*t*) uniformly over the length of this branch. Depending on the date of the duplications, the probabilities of the several resulting adjacencies are more or less linked. Hence, the integrated transition probability is no longer from one adjacency to another adjacency, but from one adjacency to the set of all the possible adjacencies that result from the duplication. In the previous example (one duplication), the transition probability is from *i*_1_*i*_2 _to ($\left(a_{\text{1}}a_{\text{2}},a_{\text{1}}a_{2}^{\prime }\right)$. We can fully model such a process as several processes in parallel. If *Q *is the generator of the binary model, *Q *⊗ *Q *⊗ ... ⊗ *Q *is the generator of the whole process, where ⊗ is the Kronecker product. Here, from a single *Q *generator at the beginning of the branch, along the branch each event of duplication gives rise to a larger Kronecker product. From a computational point of view, the whole parallel process is considered all along the branch, but just a subset of the transition probabilities is used.

We restrict here the description of the model to the case when there is at most one duplication node between two speciation nodes in the gene trees, which means that in the tree of possible adjacencies, duplication nodes have at most four descendants (because a gene duplication would have occurred in each gene tree). However, in case of several duplications, the same principle holds, with much more complicated formula.

#### One duplication

If there is one duplication in one gene tree (from *a *to *a*_1 _and *a*_2_) and no duplication in the other, then in the non duplicated branch probabilities are settled with the matrix *P*. The duplicated branch has a length drawn from the uniform distribution on the non duplication branch length, because it starts from the duplication. So the average transition matrix on the duplicated branch is:

(3) $$N^{\text{1}}\left(t\right)=\frac{1}{t}\int_{0}^{t}P\left(\tau \right)d\tau$$

As in the duplicated branch there is no adjacency (state 0) at the moment of the duplication, we are only interested by the (0, *z*) components of *N*^1^(*t*), *z *∈ [0, 1]. Calculating the integral yields:

(4) $$N_{0,0}^{1}\left(t\right)=\frac{\kappa -\kappa {e^{-}}^{\lambda t}+\lambda t}{\left(\kappa +1\right)\lambda t}$$

(5) $$N_{0,1}^{1}\left(t\right)=\frac{\kappa {e^{-}}^{\lambda t}-\kappa +\kappa \lambda t}{\left(\kappa +1\right)\lambda t}$$

Let *x *be the state of adjacency *i*_1_*i*_2_, *y *the state of *a*_1_*a*_2 _and *z *the state of $a_{\text{1}}a_{2}^{\prime }$, (*x*, *y*, *z*) ∈ [0, 1]^3^. Assuming that $a_{\text{1}}a_{2}^{\prime }$ is on the duplicated branch, the overall transition probabilities from *x *to *y *and *z *are given by $P_{x,y}\left(t\right)\times N_{0,z}^{1}\left(t\right)$.

The two choices for the duplicated branch are considered during the computation of the likelihood.

#### Two duplications

If both *i*_1 _and *i*_2 _are duplicated, we assume that both duplications are independent. Note that with this assumption, we do not model the case of joint duplications, where a fragment of chromosome is duplicated (i.e. several consecutive genes are duplicated following a single duplication event). Without loss of generality, we assume in the computation that one duplication occurs after the other. The average transition matrix integrated uniformly along both branches is:

(6) $$N^{\text{11}}\left(t\right)=\frac{2}{t^{2}}\int_{u=0}^{t}P\left(u\right)⊗\int_{v=0}^{u}P\left(v\right)⊗P\left(v\right)dvdu$$

**Figure 3:**
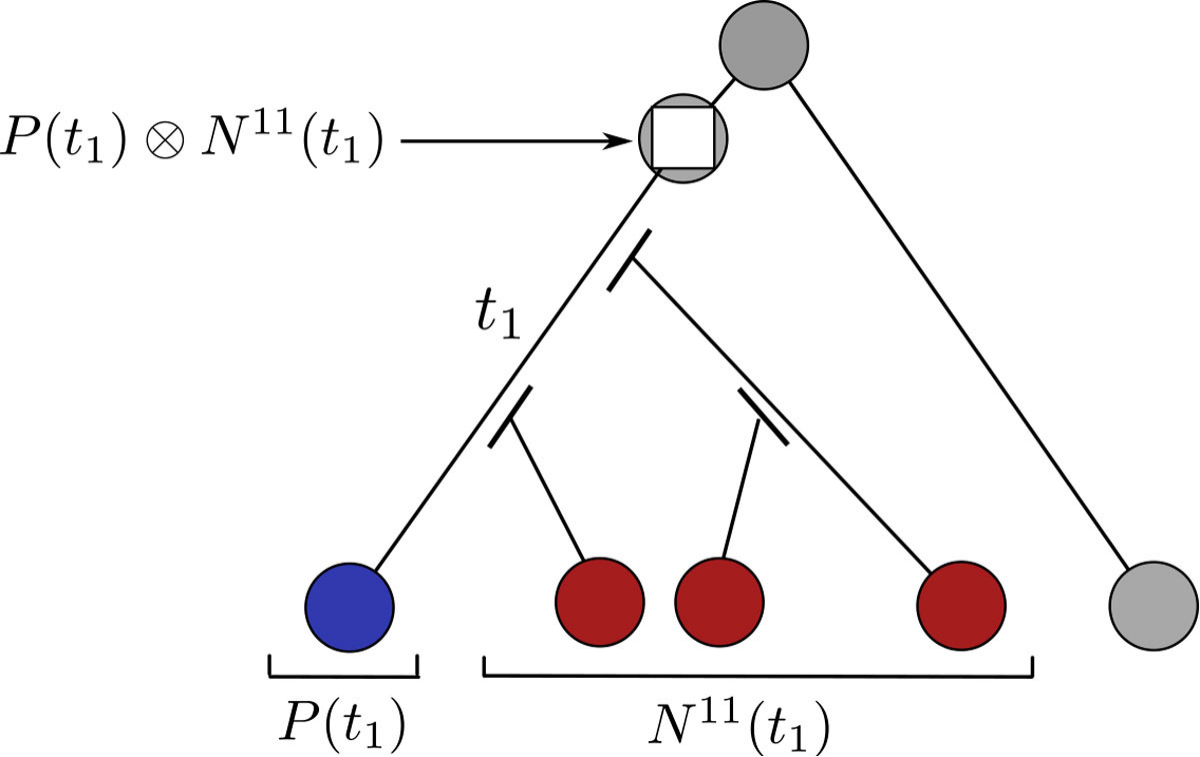
**Model of evolution with duplications**. A duplication in the same species in each of the two gene trees leads to a duplication node with four children (white square) in the tree of possible adjacencies between the two gene trees. Immediately after a duplication event, the adjacency is broken for the duplicated branch (branch leading to the rightmost red node). The second duplication leads to the simultaneous apparition of two other branches (leading to left and middle red nodes). The adjacency is also broken at the beginning of these two branches. The probabilities of transition between the duplication node and its four children are then given by the (., 0, 0, 0) → (., ., ., .) components of *P*(*t*_1_) ⊗ *N*^11^(*t*_1_). In the likelihood computation, all positions for the blue and red nodes are considered.

Since, as before, only one gene pair inherits the adjacency, we are only interested by the (., 0, 0, 0) → (., ., ., .) components of *P*(*t*) ⊗ *N*^11^(*t*) (Figure 3).

### Likelihood computation

Likelihood is computed in the rooted tree of possible adjacencies in a bottom-up way. From here, we describe adjacency nodes with single letters for better clarity. We denote as *D_i _*the data that is below node *i*.

Take a speciation node *i *in the tree, which descendants are nodes *j *and *k *(with branch lengths respectively *t*_1 _and *t*_2_). Let *x, y, z *∈ [0, 1] be the respective states of *i, j, k*. We compute the partial conditional likelihoods of *D_i _*in the classical way:

(7) $$L\left(D_{i}|x\right)=\left(\overset{1}{\underset{y=0}{\sum }}P_{xy}\left(t_{\text{1}}\right)L\left(D_{j}|y\right)\right)\cdot \left(\overset{1}{\underset{z=0}{\sum }}P_{xz}\left(t_{\text{2}}\right)P\left(D_{k}|z\right)\right)$$

Now, let *i *be a duplication node with two children *j *and *k*. Since it concerns only one branch in the species tree, there is a unique branch length *t *involved. We defined the model of evolution such that the contribution of one child is included using the basic transition matrix *P*(*t*) and the contribution of the other child (the child on the duplicated branch) is included using the transition matrix *N*^1^(*t*). The partial likelihoods of *i *can then be computed by allowing the equal possibility that either *j *or *k *is on the duplicated branch:

(8) $$\begin{array}{c} L\left(D_{i}|x\right)=\frac{1}{2}\underset{yz}{\sum }L\left(D_{j}|y\right)P_{xy}\left(t\right)L\left(D_{k}|z\right)N_{0z}^{1}\left(t\right)\\ \;\;\;\;\;\;\;\;\;\;\;\;\;\;\;\;\;\;\;\;\;\;\;\;\;\;\;\;\;\;\;\;\;\;\;\;\;\;\;\;\;\;\;\;\;\;\;\;\;\;\;+\frac{1}{2}\underset{yz}{\sum }L\left(D_{k}|y\right)P_{xy}\left(t\right)L\left(D_{j}|z\right)N_{0z}^{1}\left(t\right) \end{array}$$

If we generalize this problem, computing the partial likelihoods of a duplication node *i *means exploring the combinatorics of possible states for *i*'s children and the combinatorics of attributing the duplicated branch(es) to the children. Take a duplication node *i *with *n *speciation nodes as descendants in the same species. Each node is in a binary state, which means that there are 2*^n ^*combinations of states for *i*'s children. We could explore all these combinations to compute *i*'s likelihood but binary characters quickly lead to redundancies in the computation. We can avoid some of these redundancies and reduce the space of exploration by defining *patterns*. A *pattern *is an unordered set of 0s and 1s. There are *n*+1 possible patterns representing the states of *i*'s children. For each pattern *p*, we can compute the pattern's pseudo likelihood by exploring all its possible orders (i.e. all the possible ways of ordering the 1s and 0s in the pattern):

(9) $$L\left(D_{i}|p\right)=\underset{Y}{\sum }\underset{c\leq n}{\prod }L\left(D_{c}|Y_{c}\right)$$

where *Y *is one possible order of *p*. If *i *has *n *children, *Y *is a vector of *n *binary characters representing the states of the *n *children. *Y_c _*is thus the *c^th ^*element of *Y *and *D_c _*the data below the *c^th ^*child of *i*.

We define the *weight ω*(*p*) of the pattern *p *as the number of possible orders for $p:\omega \left(p\right)=\left(\begin{array}{c} n\\ N \end{array}\right),$ with *N *the number of 1s in *p*. We give the generalized formula for computing the partial likelihood of *i *when *i *has four children (*n *= 4, which means that the only concerned integrated transition matrix is *N *^11^(*t*)):

(10) $$L\left(D_{i}|x\right)=\underset{p}{\sum }\frac{\omega_{p}}{2^{n}}(L\left(D_{i}|p\right)\sum_{Y\in p}{\left(P⊗N^{11}\right)}_{(x,0,0,0)\to Y}(t)$$

This formula is valid for any number of children for the duplication node *i*, provided *N *^11 ^is replaced by an appropriate matrix.

### Ancestral adjacencies reconstruction

Ancestral states, that is, posterior probabilities of presence of adjacencies in the tree of possible adjacencies, are reconstructed by a top-down (from the root to the leaves) algorithm following the the bottom-up likelihood computation algorithm. In the top-down likelihood computation algorithm, we compute the conditional likelihoods of each node *i *according to the conditional likelihood of the data below it (*D_i_*), and to the conditional likelihood of the data that is on the other part of its father *f, D_f _*, and to the conditional likelihood that is below the brothers of *i *(say one brother *i*′).

If father *f *of node *i *is a speciation node:

(11) $$L\left(D|y\right)=L\left(D_{i}|y\right)\overset{1}{\underset{x=0}{\sum }}P_{xy}\left(t\right).L\left(D_{f}|x\right)\;.\overset{1}{\underset{z=0}{\sum }}P_{xz}\left(t^{\prime }\right)L\left(D_{i^{\prime }}|z\right)$$

where *y *is the state of *i, x *is the state of *f, z *the state of *i′ *and *t′ *the length of the branch from *f *to *i′*.

If father *f *of node *i *is a duplication node with one duplication (i.e. two sons *i *and *i′*), the likelihood of node *i *is the average of both scenarios:

(12) $$L\left(D|y\right)=L\left(D_{i}|y\right).\frac{1}{2}\overset{1}{\underset{x=0}{\sum }}L\left(D_{f}|x\right).\overset{1}{\underset{z=0}{\sum }}\left(P_{xy}\left(t\right)N_{0z}^{1}\left(t^{\prime }\right)+P_{xz}\left(t^{\prime }\right).N_{{}_{0y}}^{1}\left(t\right)\right)L\left(D_{i^{\prime }}|z\right)$$

And the equivalent to the case of two duplications in the bottom-up algorithm is achieved by computing *i*'s partial likelihoods when *i*'s father is a duplication node with four children *i, i′, i″, i‴*, and the likelihood is an average of four scenarios:

(13) $$\begin{array}{c} L\left(D|y\right)=L\left(D_{i}|y\right).\frac{1}{4}\overset{1}{\underset{x=0}{\sum }}L\left(D_{f}|x\right).\\ \;\underset{wzu}{\sum }(P_{xy}\left(t\right).N_{0,0,0\to w,z,u}^{11}\left(t\right)+P_{xw}\left(t\right)N_{0,0,0\to y,z,u}^{11}\left(t\right)\\ \;+P_{xz}\left(t\right)N_{0,0,0\to w,y,u}^{11}\left(t\right)+P_{xu}\left(t\right)N_{0,0,0\to w,z,y}^{11}\left(t)\right)\\ \;L\left(D_{i^{\prime }}|w\right).L\left(D_{i^{\prime \prime }}|z\right).L\left(D_{i^{\prime \prime \prime }}|u\right) \end{array}$$

From these conditional likelihoods, *a posteriori *probabilities of presence of adjacencies can be computed. The result is, for each ancestral species, a set of adjacencies associated with probabilities of presence. Transforming it into a *bona fide *gene order necessitates finding a subset of probable adjacencies in which one ancestral gene can be adjacent to only two others. Efficient methods exist [20] to do so, but they ignore the main source of possible conflict between adjacencies when they are seen as independently evolving characters: errors in gene trees [27]. So in general we prefer presenting a set of adjacencies associated with probabilities, and leave open the way of choosing among them and/or correcting the input data to avoid conflict.

### Implementation and availability

We implemented the model of evolution and the likelihood calculation algorithm in the Bio++ library (http://biopp.univ-montp2.fr/). The algorithm that builds the trees of possible adjacencies was implemented in a separate program which also uses Bio++. Reconciliation was performed with DeCo [10]. All the analytical formulas in our model were computed using Maxima (see Additional file 1). These programs are available upon request to the authors.

## Results

### Dataset

**Figure 4:**
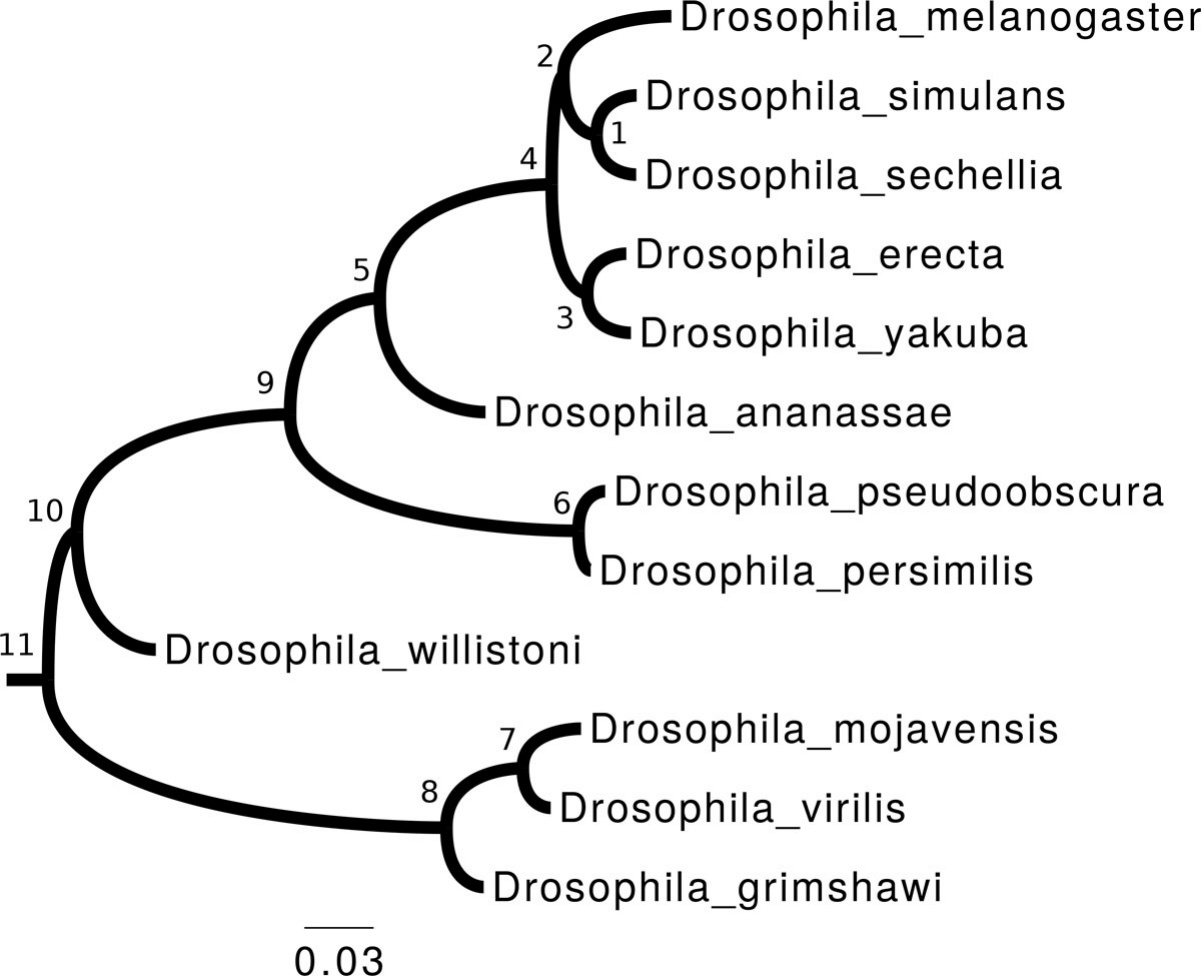
**Drosophila phylogeny**. The 12 Drosophila species tree with branch lengths optimized according to the model and the synteny data.

We selected 12 drosophila species from the Ensembl Metazoa [24] database. We used the species tree from [28], the gene trees and the chromosomal locations from Ensembl Metazoa. We pruned the gene trees to keep only the drosophilae clade, and reconciled them with the species tree using [10]. We reduced the dataset to the 9223 gene trees with at most one duplication between two speciation nodes in reconciled gene trees. We built a set of extant adjacencies by connecting consecutive genes in the reduced dataset, provided they were on the same chromosome or scaffold. We built 13059 trees of possible adjacencies from this set of reconciled gene trees and extant adjacencies. By maximum likelihood, we optimized the branch lengths of the species tree using our model of evolution, from the 3608 trees of possible adjacencies without any duplication (Figure 4). Optimizing branch lengths over many trees remains computationally intensive, especially for trees with several duplications (then the combinatorics increases). The choice of the sample to optimize from was thus a trade-off between accuracy and computational cost. While we optimized branch lengths, we also optimized the model's parameters in a non-stationary way.

Note that the drosophila genomes are not all perfectly assembled and some are fragmented in several hundred contigs. So all the signal does not have to be interpreted as rearrangements, but some of it is due to the absence of adjacencies in extant genomes.

### Ancestral adjacencies

We computed posterior probabilities of presence and absence for all possible ancestral adjacencies, given the optimized branch lengths. We report in Table 1 the number of genes and adjacencies in extant and ancestral species. Note that the difference between the number of genes and adjacencies in extant species gives the number of chromosomes or scaffolds. This goes from the well assembled *melanogaster *genomes in 8 scaffolds to *simulans *with 445 scaffolds, with all intermediaries. Despite the fact that assembly is incomplete, we have enough adjacencies in the dataset to make a signal for the reconstruction of ancestral adjacencies. And indeed, 54222 adjacencies with posterior probability > 0.9 are proposed. The signal is weaker for ancient species, as in ANC10, with only 2360 adjacencies for 8026 genes, depicting a very fragmented ancestral genome.

**Table 1:** Statistics of extant and ancestral genomes in the drosophila dataset.

Extant species	genes	adjacencies	coverage
*melanogaster*	6410	6402	47%
*simulans*	7195	6750	50%
*sechellia*	7551	7261	48%
*erecta*	6961	6910	49%
*yacuba*	7313	7058	49%
*ananassae*	6558	6459	47%
*pseudoobscura*	7280	7007	48%
*persimilis*	7361	7025	47%
*willistoni*	6236	6063	43%
*mojavensis*	6484	6403	48%
*virilis*	6512	6437	48%
*grimsawi*	6538	6220	46%

**Ancestral species**	**genes**	**adjacencies > 0.9**	**genes with more than 2 adjacencies**

ANC1	8054	7164	578
ANC2	8364	5422	164
ANC3	8696	7529	1348
ANC4	9455	3746	113
ANC5	7564	5021	160
ANC6	7242	6117	58
ANC7	6677	6184	210
ANC8	6954	5777	413
ANC9	8816	2872	47
ANC10	8026	2360	24
ANC11	7157	2030	3

Column "genes" is the number of genes in the dataset. Column "coverage" is the proportion of the genes in the dataset to the total number of genes annotated in Ensembl. Column "adjacencies" is the number of adjacencies in an extant genome or adjacencies in ancestral genomes with a posterior probability > 0.9. Column "genes with more than 2 adjacencies" is the number of genes involved in more than 2 adjacencies of posterior probability > 0.9. This value is reported only for ancestral genomes, as in extant all genes have 0, 1 or 2 neighbors by definition.

The "degree" column in Table 1 shows that in general less than 4% of the genes harbor a conflicting signal with more than 2 attached adjacencies having posterior probability > 0.9. While this remains a high rate of error, it means that most of the supported signal constitutes linear ancestral contigs or chromosomes. The conflict is variable according to the lineages. A surprisingly high amount of conflict arises for the ancestor of *yacuba *and *erecta*, predicted as recent. Perhaps this reflects an ambiguity in the species tree which precisely at this place is debated [29]. It seems that rearrangements support an alternative topology.

### Comparison with parsimony

**Figure 5:**
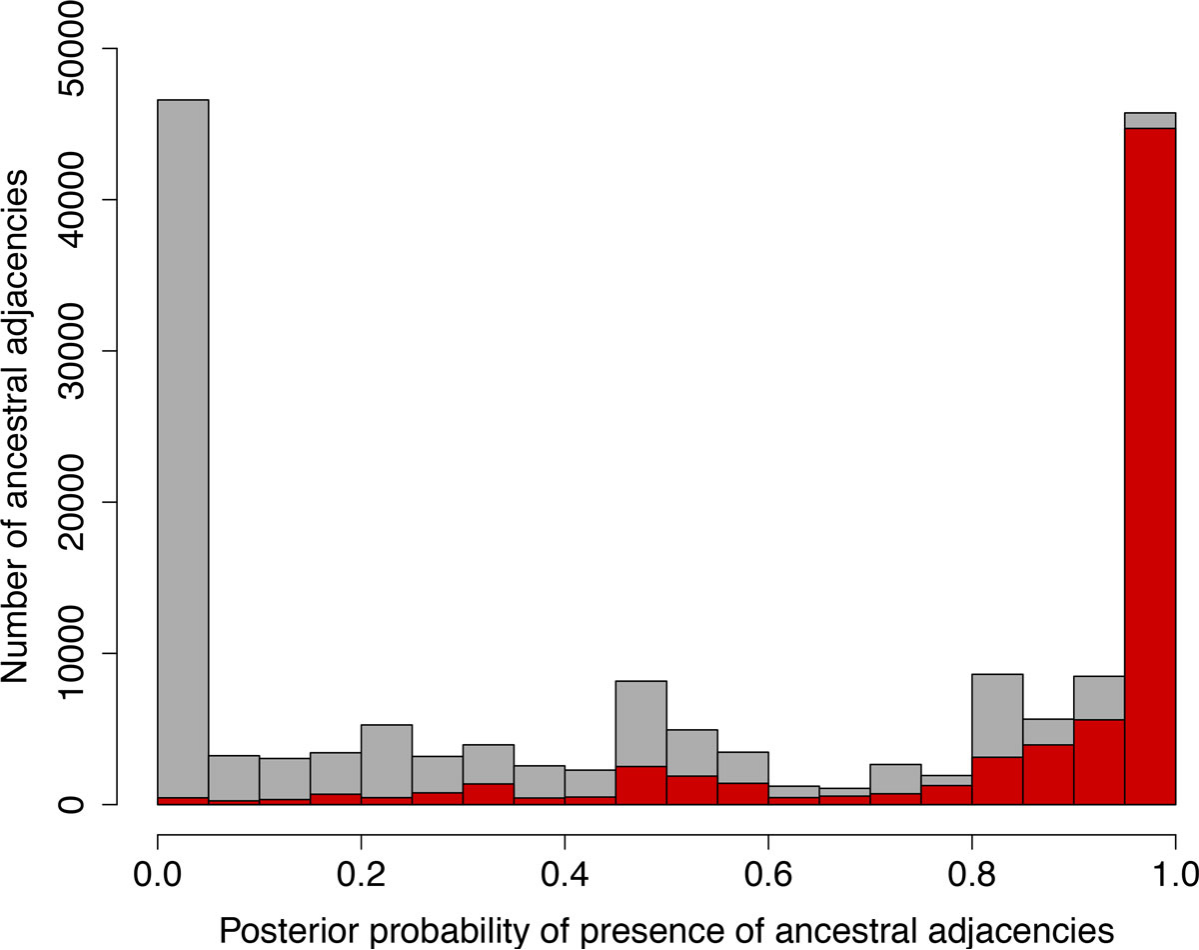
**Ancestral adjacencies reconstruction with our method and with DeCo**. The posterior probabilities of presence of ancestral adjacencies reconstructed with our model (in grey). In red the part reconstructed by a parsimony method.

We compare the results with those obtained by [10] (DeCo software) on the same data (Figure 5). DeCo reconstructs ancestral adjacencies according to a parsimony principle, whereas we reconstruct all possible ancestral adjacencies along with a posterior probability of presence for each one. Most of the adjacencies reconstructed by DeCo are given a high probability of presence according to our model (70% have a support > 0.9). Interestingly, a few of them are given low probabilities of presence (11% have probabilities of presence < 0.5), suggesting that our model could bring a finer understanding of the evolution of these adjacencies. Figure 5 shows the distribution of posterior probabilities, as computed by our model, of all the possible adjacencies (in grey), and of all the adjacencies inferred by parsimony (in red).

We always reconstruct more ancestral adjacencies than DeCo because DeCo reconstructs ancestral adjacencies up to the last common ancestor of an adjacency class, whereas we reconstruct possible ancestral adjacencies up to the most ancient ancestor of an adjacency class. This explains why many possible ancestral adjacencies have low or no support in the presence/absence pattern at the leaves.

## Discussion

Probabilistic models of evolution have at least four advantages over parsimony approaches: they provide more accurate results in presence of many mutations; they provide a natural support scheme of the results in the form of a probability of ancestral states; the likelihood is computed by an integration over all scenarios rather than choosing only one, even if optimal; and several models at different scales of the genome can be integrated.

But most probabilistic models of gene order evolution are computationally intractable on large datasets, working with too large state spaces. Coding gene order by binary characters is a solution, like for many characters characterized by their presence or absence. Then it is possible, like in [30], to use a standard model of binary sequence evolution to achieve a probabilistic reconstruction of phylogenies and ancestral gene orders based on the presence/absence of adjacencies in extant species. This way can handle unequal gene content but does not model the processes of joint evolution of gene content and order, and has to simplify the data to make it fit into standard models. As a result a part of the understanding of genome evolution remains out of reach.

This is why we put some efforts in a model of gene neighborhood evolution handling complex histories of genes depicted by their reconciled phylogenies.

We gain several advantages. For example the model allows to follow a pattern of descent of adjacencies. Links between genes evolve, just as genes evolve too. This can be used to detect the positional orthology (orthology of a gene as a locus, in addition to a sequence) when a gene is duplicated in an asymmetric way [31] - not in tandem, so that from the loci point of view, only one duplicate is a descendant of the unique copy before duplication. Here we allow any kind of duplication, symmetric or not, but in any case an adjacency is transmitted to one copy. In the case of a tandem duplication, this does not yield an asymmetry for the genes, because a gene has two adjacencies, and the two can transmit a descendant to a different copy in the case of a tandem duplication. But in the case of an asymmetric duplication, the two adjacencies are transmitted to the same copy of a gene and a positional homolog is detected.

We also keep track of the evolutionary events that can be responsible for the gain and loss of an adjacency. For example an adjacency can be lost because one of the genes is lost, or because of a rearrangement. It is two different reasons for an adjacency to be absent, and we are able with a model to differentiate both cases.

We found that a significant number of adjacencies inferred by parsimony on a drosophila dataset are not supported by a probabilistic model. It corroborates the usual findings in evolutionary models each time reasonably distant species are compared, whether it is sequence evolution [1], gene content evolution [32], or gene order evolution [12].

There are still several limitations to this work. For the moment the computation time is one of them, the efficiency of optimization algorithms coupled with our model allowed us to work only on a small fixed phylogeny. Theoretically we could even infer phylogenies, coupling a model of sequence evolution and such a model of genome organization evolution, but it will necessitate algorithmic progresses. Another limit is that our current implementation only handles independent duplication events, although we are also developing a model for joint duplications. Finally, the possible presence of many duplications yields intricate integrals difficult to solve analytically, if we want to stick with exact solutions integrating over their position in a branch. Numerical approximations or simplifying hypotheses have to be incorporated. For the moment families with many duplications are filtered out.

## Conclusions

The present model is a proof of concept that it is possible to handle whole genomes of dozens of species, including genes with complex histories, into a probabilistic model for gene organization.

We open a path that has many possible continuations:

• Handle joint duplications of two consecutive genes as a single duplication event.

• Handle more than one gene duplication between two gene speciations.

• Handle horizontal gene transfer (a parsimonious framework is available [33]).

• Jointly infer probabilistic presence and absence of genes and gene neighborhoods, using conditional probabilities mixing two models.

• Integrate the model into an integrative probabilistic model of genome evolution, handling both sequence evolution and gene content evolution, like Phyldog [27].

• With this integration the model can be used to infer species phylogenies, or at least in the current state of computational complexity, to test among a small number of species phylogenies. For example we will test two different alternative drosophila species tree topologies according to the likelihood of our model, and according to the coherence of ancestral genomes (the linear organization of genes along chromosomes).

• Use this model to detect highly variable sites by correlating variable rates of adjacency evolution (in a similar framework as for sequence evolution [34]) and intergene sizes, and bring a stone to the study of fragile and solid regions [26].

These constitute our work in progress. We see the model we present here as a decisive step.

## Competing interests

The authors declare that they have no competing interests.

## Authors' contributions

MS, ET and LG wrote the model, MS and LG implemented it and MS did the experiments.

## Supplementary Material

Additional file 1 **Maxima commands for the model of evolution**. Click here for file
