# Supplementary material for: Probabilistic modeling of the evolution of gene synteny within reconciled phylogenies
Source: BMC Bioinformatics. 2015 Oct 2;16(Suppl 14):S5. doi: 10.1186/1471-2105-16-S14-S5 (PMC4603630; doi:10.1186/1471-2105-16-S14-S5)
Supplement: Additional file 1 — Maxima commands for the model of evolution. [file 1471-2105-16-S14-S5-S1.pdf]

Maxima commands for the model of evolution

Magali Semeria, Eric Tannier, Laurent Guéguen

July 20, 2015

All the analytical formulas in our model were computed using Maxima (http://maxima.sourceforge.net). We provide here the commands used to obtain the transition probabilities for one duplication and two duplications. We denote

$$P(t) = \begin{pmatrix} \frac{k\, e^{-\frac{(k+1)^2\, r\, t}{2\, k}}+1}{k+1} & \frac{k \left(1-e^{-\frac{(k+1)^2\, r\, t}{2\, k}}\right)}{k+1} \\ \frac{1-e^{-\frac{(k+1)^2\, r\, t}{2\, k}}}{k+1} & \frac{e^{-\frac{(k+1)^2\, r\, t}{2\, k}}+k}{k+1} \end{pmatrix}$$

the basic transition matrix in time t.
Maxima commands:

```
 P: matrix([(1+k*exp(-(k+1)^2/(2*k)*r*t))/(k+1),
k*(1-exp(-(k+1)^2/(2*k)*r*t))/(k+1)],
[(1-exp(-(k+1)^2/(2*k)*r*t))/(k+1),
(k+exp(-(k+1)^2/(2*k)*r*t))/(k+1)]);
```

One duplication

The average transition matrix integrated along the duplicated branch is

$$N_1(t) = \frac{1}{T} \int_0^T P(\tau) d\tau$$

and the overall transition probabilities are the $(x, 0) \rightarrow (y, z)$ components of $P(t) \otimes N_1(t)$.
Maxima commands:

```
assume(T>0);

IN1 : matrixmap(lambda([x],multthru(integrate(x,t,0,T))),P)$

N1 : matrixmap(lambda([x],multthru(x/T)),IN1)$

tex(N1)$
```

- Transition probabilities of $N_1(t)$ from state 0:

$$N_1(t)(0,0) = \frac{\frac{k-k\,e^{-T\,l\,r}}{T\,l\,r}+1}{k+1}$$
$$N_1(t)(0,1)= \frac{k\,e^{-l\,r\,T}-k}{(k+1)\;l\,r\,T}+\frac{k}{k+1}$$

Two duplications

The average transition matrix integrated along both branches is

$$N_{11}(t) = \frac{2}{t^2}\int_{T_1=0}^{t} P(T_1)\otimes\int_{T_2=0}^{T_1} P(T_2)\otimes P(T_2)dT_2dT_1$$

and the overall transition probabilities are the $(x,0,0,0) \rightarrow (.,.,.,.)$ components of $P(t)\otimes N_{11}(t)$.
Maxima commands:

```
PkP : kronecker_product(P,P)$

assume(T>0);

iPkP : matrixmap(lambda([x],multthru(integrate(x,t,0,T))),PkP)$

iPkP_t : iPkP, T=t$

PkiPkP : kronecker_product(P, iPkP_t)$

N11 : matrixmap(lambda([x],multthru(2*integrate(x,t,0,T)/T^2)),PkiPkP)$

tex(N11)$
```

- Transition probabilities from state (0,0,0):

$$N_{11}[(0,0,0),(0,0,0)] = \frac{\left(-k^2-4\,k+2\right)\,e^{-l\,r\,T}+\frac{5\,k\,e^{-2\,l\,r\,T}}{2}+\frac{k^2\,e^{-3\,l\,r\,T}}{3}+\frac{4\,k^2+9\,k-12}{6}}{2\;(k+1)\;l^3\,r^2\,T^2} + \frac{\frac{k+4}{2}-e^{-l\,r\,T}}{(k+1)\;l^2\,r\,T}+\frac{1}{(k+1)^3}$$

$$N_{11}[(0,0,0),(0,0,1)] = \frac{\left(-\,(k+1)^2\;r\,T-(k-1)^2-1\right)\;e^{-l\,r\,T}+\frac{k\,(2\,k-3)\,e^{-2\,l\,r\,T}}{2}-\frac{k^2\,e^{-3\,l\,r\,T}}{3}+\frac{2\,k^2-3\,k+12}{6}}{2\;(k+1)\;l^3\,r^2\,T^2} + \frac{k-2}{2\;(k+1)\;l^2\,r\,T}+\frac{k}{(k+1)^3}$$

$$N_{11}[(0,0,0),(0,1,0)] = N_{11}[(0,0,0),(0,0,1)$$

$$N_{11}[(0,0,0),(0,1,1)] = \frac{-k\,\left((k+1)^2\; r\,T - k + 4\right)\, e^{-l\,r\,T} - \frac{k\,(4\,k-1)\,e^{-2\,l\,r\,T}}{2} + \frac{k^2\,e^{-3\,l\,r\,T}}{3} + \frac{k\,(4\,k+21)}{6}}{2\;(k+1)\;l^3\,r^2\,T^2} - \frac{3\,k}{2\;(k+1)\;l^2\,r\,T} + \frac{k^2}{(k+1)^3}$$

$$N_{11}[(0,0,0),(1,0,0)] = \frac{\left(k^2 + 8\,k + 2\right)\, e^{-l\,r\,T} + \frac{(k-4)\,k\,e^{-2\,l\,r\,T}}{2} - \frac{k^2\,e^{-3\,l\,r\,T}}{3} + \frac{-7\,k^2 - 36\,k - 12}{6}}{2\;(k+1)\;l^3\,r^2\,T^2} + \frac{e^{-l\,r\,T} + \frac{k\,(k+4)}{2}}{(k+1)\;l^2\,r\,T} + \frac{k}{(k+1)^3}$$

$$N_{11}[(0,0,0),(1,0,1)] = \frac{\left((k+1)^2\; r\,T + k\;(3\,k-2)\right)\, e^{-l\,r\,T} - \frac{k\,(3\,k-2)\,e^{-2\,l\,r\,T}}{2} + \frac{k^2\,e^{-3\,l\,r\,T}}{3} - \frac{k\,(11\,k-6)}{6}}{2\;(k+1)\;l^3\,r^2\,T^2} + \frac{(k-2)\;k}{2\;(k+1)\;l^2\,r\,T} + \frac{k^2}{(k+1)^3}$$

$$N_{11}[(0,0,0),(1,1,0)] = N_{11}[(0,0,0),(1,0,1)]$$

$$N_{11}[(0,0,0),(1,1,1)] = \frac{k\,\left((k+1)^2\; r\,T - 5\,k\right)\, e^{-l\,r\,T} + \frac{5\,k^2\,e^{-2\,l\,r\,T}}{2} - \frac{k^2\,e^{-3\,l\,r\,T}}{3} + \frac{17\,k^2}{6}}{2\;(k+1)\;l^3\,r^2\,T^2} - \frac{3\,k^2}{2\;(k+1)\;l^2\,r\,T} + \frac{k^3}{(k+1)^3}$$
